# Screening for periodontal disease in research dogs - a methodology study

**DOI:** 10.1186/s13028-014-0077-8

**Published:** 2014-11-19

**Authors:** Hanne E Kortegaard, Thomas Eriksen, Vibeke Baelum

**Affiliations:** Department of Veterinary Clinical and Animal Sciences, Faculty of Health and Medical Sciences, University of Copenhagen, Dyrlægevej 16, DK-1870 Frederiksberg C, Denmark; Department of Dentistry, Aarhus University, Vennelyst Boulevard 9, DK-8000 Aarhus C, Denmark

**Keywords:** Periodontal disease, Clinical attachment loss, Pocket depth, Dogs, Screening

## Abstract

**Background:**

It has been shown that the prevalence of both clinical attachment loss (CAL) ≥1 mm and pocket probing depth (PPD) ≥4 mm is relatively high even in younger dogs, but also that only a minority of the dogs have such clinical signs of periodontal disease (PD) in more than a few teeth. Hence, a minority of dogs carry the major PD burden. These epidemiological features suggest that screening for PD in larger groups of dogs, allowing for rapid assessment of treatment planning, or for the selection of dogs with or without PD prior to be included in experimental trials, should be possible. CAL is the central variable in assessing PD extent and severity while PPD is the central variable used in treatment planning which make these two variables obvious in a screening protocol with the dual aim of disease identification and treatment planning. The main purpose of the present study in 98 laboratory Beagle dogs was to construct a fast, simple and accurate screening tool, which is highly sensitive for the identification of dogs with PD.

**Results:**

Examination of the maxillary P4, P3, P2, I1 and C would, in this population, result in the identification of 85.5% of all dogs and 96% of all teeth positive for CAL ≥1 mm, and 58.9% of all dogs and 82.1% of all teeth positive for PD ≥4 mm.

Examination of tooth pairs, all C’s, maxillary I2, M2 and the mandibular P4 would, in this population result in identification of 92.9% of all dogs and 97.3% of all teeth positive for PD ≥4 mm, and 65.5% of all dogs and 83.2% of all teeth positive for CAL ≥1 mm. The results presented here only pertain to the present study population.

**Conclusions:**

This screening protocol is suitable for examination of larger groups of laboratory Beagle dogs for PD and our findings indicate that diseased dogs are identified with a high degree of sensitivity. Before this screening can be used in clinical practice, it has to be validated in breeds other than Beagle dogs and in populations with larger age variation.

## Background

Identification of dogs with periodontal disease (PD) is important in order to maintain oro-dental health and function and to assess periodontal destruction and the related risk of systemic complications [1-5]. The typical reason for anesthetizing dogs for periodontal examination is poor oral hygiene and halitosis, for which the main treatment is thorough dental scaling and polishing as well as extractions of severely diseased teeth; however, advanced periodontal treatment including subgingival curettage, surgery and alveolar bone regeneration is possible. Beagle dogs, living in experimental facilities, are in the same need of regular dental care as pet dogs. An increasing number of studies in humans indicate that also periodontal disease is associated with systemic disorders, especially cardiovascular disease [6-8]. A correlation between PD and e.g. elevated serum C reactive protein has been identified and linked to the development of atherosclerosis [9]. PD related systemic complications may present as a periopathogenic bacteremia and subsequent distant (focal) infections; as periopathogenic toxemia or as a systemic immune mediated reaction induced by the localized periodontal inflammatory lesion [7]. Assessment of the periodontal status of dogs both for clinical and experimental purposes includes assessment of dental deposits, degree of inflammation and attachment loss. This includes a full mouth, site-specific evaluation of the amounts of plaque and calculus present, the gingival inflammatory status, as well as estimation of the clinical attachment level (CAL) and the probing pocket depth (PPD), supplemented by a radiographic assessment of the alveolar bone level of all 42 teeth [10-14]. However, this diagnostic procedure is rather time consuming even for a trained practitioner and anaesthesia time may therefore become undesirably long.

In a previous study of Beagle dogs [15] we have shown that the prevalence of both clinical attachment loss (CAL) ≥1 mm and pocket probing depth (PPD) ≥4 mm are relatively high even in younger dogs, but also that only a minority of the dogs have such clinical signs of PD in more than a few teeth. Hence, a minority of dogs carry the major PD burden. These epidemiological features suggest that screening for PD in larger groups of dogs, allowing for rapid assessment of treatment planning, or for the selection of dogs with or without PD prior to be included in experimental trials, should be possible. CAL is the central variable in assessing PD extent and severity while PPD is the central variable used in treatment planning which make these two variables obvious in a screening protocol with the dual aim of disease identification and treatment planning.

A number of studies suggest that PD among dogs [15-18], in corroboration with PD in humans [19-21], follows a particular pattern within the dentition, which may be utilized to develop a tool for screening dogs for PD. A total periodontal disease index for disease estimation has been described; however this index aims at estimating disease extent within a dentition, not as in this present study disease occurrence within a population [22,23].

The main purpose of the present study was to construct a fast, simple and accurate screening tool, which is highly sensitive for the identification of dogs with PD.

## Methods

The data used for the present study were extracted from a previous study [15]. Briefly, 98 clinically healthy Beagle dogs (57 females and 41 males) aged between 13 months and 7 years (mean age 32.4 months; SD 15.7 months) and with a mean weight of 15 kg; SD 2.2 kg) were examined as part of a routinely performed health-monitoring programme. The dogs originated in two research facilities that house Beagle colonies. The dogs were mainly fed a diet consisting of commercial dry pellets and water ad libitum. None of the dogs had had any dental prophylaxis or treatment performed within the last year and the status of the dogs’ periodontal health and oral hygiene was unknown prior to examination. The protocol for this study was reviewed and approved by the Internal Animal Care and Use Committee for the Veterinary Teaching Hospital and by the National Animal Ethics Council.

### Clinical examination procedures

**Figure 1:**
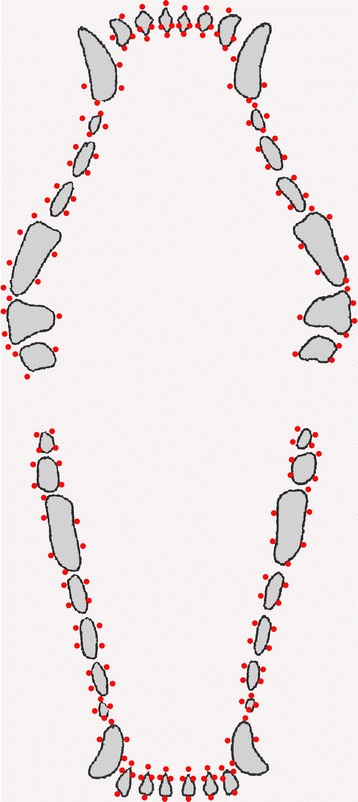
**Occlusal view of a dog dentition.** Occlusal view of the canine dentition marked with the specific sites examined.

All dogs were premedicated with either propionyl promazine (0.05 mg/kg) and atropine (0.03 mg/kg) intramuscularly (im) or diazepam (0.4 mg/kg) and atropine (0.03 mg/kg) im. Anaesthesia was induced with propofol (4 mg/kg) intravenously (iv) and maintained by continuous iv infusion of propofol (0.35 mg/kg/minute) using a syringe pump (Terumo Terufusion Syringe Pump; Hemax Medicals APS, Hvidovre, Denmark). All dogs received a full mouth examination, which included the examination of all teeth for CAL, as the distance in mm from the cemento-enamel-junction to the bottom of the pocket using a periodontal probe (LM 23-52B XSi; LMDental, Finland), measured in 3 to 6 sites per tooth depending on the tooth type (Figure 1). Measurements were rounded to the nearest lower value in mm. PPD was measured in mm as the distance from the gingival margin to the bottom of the pocket in the same 176 sites. Deposits were removed only if their presence made it impossible to measure CAL or PPD. All dogs were examined by the same examiner. Recordings of CAL were repeated for 5 randomly selected teeth in each of 25 dogs for the purpose of assessing the intra-examiner reliability [15].

### Data analysis

Tooth specific CAL and PPD values were generated by selecting among the site-specific recordings as the most severe recording for each tooth present, irrespective of site location of the recording. Frequency distributions were subsequently generated of the distribution of CAL and PPD within the dentition [15]. All teeth in pairs left to right (a total of 21 tooth types) were considered. Tooth subsets to be included in the screening system were identified by the following algorithm. First, the tooth type most frequently affected with CAL ≥1 mm or PPD ≥4 mm was selected. Animals thus affected were identified and excluded from further analysis. Subsequently, the tooth type most likely to have CAL ≥ 1 mm or PPD ≥4 mm among the dogs still retained in the analysis was identified and the dogs with a screen positive test in this tooth type were identified and excluded. This algorithm was continued until all positive dogs had been identified. At each step sensitivity values were calculated, using both the number of dogs and the number of teeth affected as the denominator. By virtue of the nature of the algorithm the ‘screen test’ specificity values will necessarily be 1 at each step. We have therefore at each step calculated the predictive value of a screen negative test (NPV) at the dog level, i.e., the probability that the dog does not have CAL (or PPD) when the test is negative.

## Results

All dogs had at least 40 teeth present, and 91% had a full dentition. The history of the missing teeth was unknown. A total of 55 dogs (56%) were positive for CAL ≥1 mm, and the proportion was higher among dogs aged 2.5 yrs. or above (78%) than among dogs younger than 2.5 yrs. (33%) (Table 1). A total of 56 dogs (57%) were positive for PPD ≥4 mm, and the proportion was higher among dogs aged 2.5 yrs. or above (74%) than among dogs younger than 2.5 yrs (40%) (Table 1). Below the age of 2.5 yrs. relatively few teeth presented with CAL ≥1 mm or PPD ≥4 mm, whereas considerably more teeth were affected among older dogs (Table 1).

**Table 1:** **Distribution of clinical attachment loss (CAL) and pocket probing depth (PPD) in teeth/dogs**

	**Clinical attachment loss CAL**				**Pocket probing depth PPD**			
	**N =48**		**N =50**		**N =48**		**N =50**	
	**< 2.5 yrs**		≥**2.5 yrs**		**< 2.5 yrs**		≥**2.5 yrs**	
**mm**	**% dogs**	**% teeth**	**% dogs**	**% teeth**	**% dogs**	**% teeth**	**% dogs**	**% teeth**
**0**	66.7	98.5	22.0	88.9				
**1**	10.4	0.7	16.0	0.9	-	46.2	-	28.8
**2**	18.8	0.8	16.0	4.0	4.2	45.1	-	47.3
**3**	2.1	0.1	6.0	2.1	56.3	7.1	26.0	14.8
**4**	2.1	0.1	22.0	2.3	31.3	1.4	32.0	6.0
**5**			8.0	0.9	8.3	0.2	24.0	1.8
**6**			6.0	0.6			12.0	0.9
**7**				0.2			4.0	0.3
**8**			4.0	0.1				0.10
**9+**							2.0	0.1

Distribution of the number of teeth and the number of dogs according to the highest recording of CAL, respectively PPD, in the tooth/dog. Given according to age of the dogs.

Table 2 shows that although the number of dogs affected by CAL ≥1 mm was essentially the same as the number of dogs with PPD ≥4 mm, the overlap between the two groups was not perfect. Similarly, although the number of teeth with CAL ≥1 mm came close to the number of teeth with PPD ≥4 mm, the teeth affected were not the same. These features indicate that the strategy for screening for CAL ≥1 mm may be rather different from the strategy employed to identify PPD ≥4 mm.

**Table 2:** **Distribution of clinical attachment loss (CAL) and pocket probing depth (PPD) amongst the dogs**

	**PPD** ≥**4 mm**	**PPD <4 mm**	
**CAL** ≥ **1 mm**	40	15	55
**CAL < 1 mm**	16	27	43
**Total no. of dogs**	56	42	98

Cross tabulation of dogs according to their diagnosis with respect to the presence, respectively absence, of CAL ≥1 mm and PPD ≥4 mm.

### Development of partial subsets for screening

**Figure 2:**
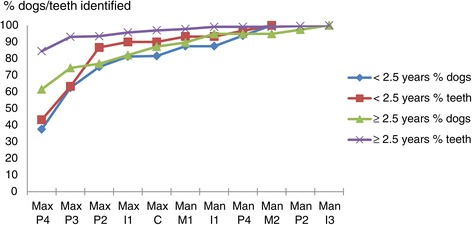
**Identification of presence of clinical attachment loss (CAL) ≥1 mm.** The portion of the total number of teeth/dogs with CAL ≥1 mm that are identified when successively examining the teeth indicated on the x-axis. Given according to age.

The teeth most likely to exhibit CAL ≥1 mm were the maxillary P4’s. Screening of these two teeth would result in identification of 54.5% of all the dogs positive for CAL ≥1 mm and 79.8% of all the positive teeth in the population (Table 3, Figure 2). Adding the maxillary P3’s and P2’s to the partial subset of teeth examined would increase the proportion of positive dogs identified to 76.4% and the proportion of positive teeth identified to 92.7%. Adding the first upper incisors to the partial subset examined raised these figures to 81.8% and 95.0%, respectively. Identification of all dogs and all teeth positive for CAL ≥1 mm required the screening of 11 of 21 possible tooth-pairs (Table 3). Figure 2 shows for both age groups that the increase in sensitivity associated with the inclusion of an additional tooth-pair to the partial subset of teeth examined was most pronounced for the maxillary P4’s, P3’s, P2’s and I1’s, and that addition of more tooth-pairs to the partial subset examined resulted in limited gains in the sensitivity of the test for the detection of CAL ≥1 mm. Screening only animals aged over 2.5 years for CAL ≥1 mm resulted in slightly higher sensitivity values than screening younger dogs (Figure 2).

**Table 3:** **Hierarchical identification of dogs with recordings of clinical attachment loss (CAL)** ≥**1 mm**

**Tooth- pair examined**	**All dogs (N = 98)**		
	**% CAL positive dogs found**	**% CAL positive teeth found**	NPV* dogs
**Max P4**	54.5	79.8	0.63
**+Max P3**	70.9	89.7	0.73
**+Max P2**	76.4	92.7	0.77
**+Max I1**	81.8	95.0	0.81
**+Max C**	85.5	96.2	0.84
**+Man M1**	89.1	97.3	0.88
**+Man I1**	92.7	98.5	0.91
**+Man P4**	94.5	98.9	0.93
**+Man M2**	96.4	99.2	0.96
**+Man P2**	98.2	99.6	0.98
**+Man I3**	100	100	1.00

The proportion of the total number of CAL positive dogs, respectively teeth that are identified by examination of the indicated tooth pairs (sensitivity). Also given for each combination of tooth-pairs is the proportion of test-negative dogs that are truly negative for the presence of CAL ≥1 mm (Negative predictive value).

*Negative predictive value of screen test at dog level.

**Figure 3:**
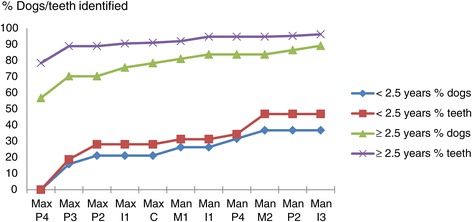
**Identification of presence of pocket probing depth (PPD) ≥4 mm using partial subset for CAL ≥1 mm.** The portion of the total number of teeth/dogs with PPD ≥4 mm that are identified when successively examining the teeth indicated on the x-axis. Given according to age.

Table 4 shows that examination of the maxillary P4’s, P3’s, P2’s and I1’s would result in the identification of 57.1% of the dogs, and 81.6% of the teeth that had PPD ≥4 mm. Again, screening only animals aged over 2.5 years of age for PPD ≥4 mm has a rather high sensitivity (Figure 3), whereas the screening of younger dogs using this subset of teeth resulted in the identification of less than half of the dogs or teeth with PPD ≥4 mm.

**Table 4:** **Presence of pocket probing depth (PPD) ≥4 mm using the screening subset for clinical attachment loss (CAL) ≥1mm**

	**All dogs (N = 98)**	
**Tooth-pair examined**	**% PPD positive dogs found**	**% PPD positive teeth found**
**Max P4**	37.5	67.3
**+Max P3**	51.8	78.9
**+Max P2**	53.6	80.3
**+Max I1**	57.1	81.6
**+Max C**	58.9	82.1
**+Man M1**	62.5	83.4
**+Man I1**	64.3	85.6
**+Man P4**	66.1	86.1
**+Man M2**	67.9	87.9
**+Man P2**	69.6	88.3
**+Man I3**	71.4	89.2

The proportion of the total number of PPD ≥4 mm positive dogs, respectively, teeth that are identified by examination of the tooth pairs indicated for identifying CAL ≥1mm (sensitivity).

**Figure 4:**
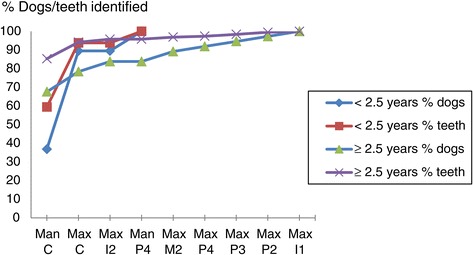
**Identification of presence of pocket probing depth (PPD) ≥4 mm.** The portion of the total number of teeth/dogs with PPD ≥4 mm that are identified when successively examining the teeth indicated on the x-axis. Given according to age.

**Figure 5:**
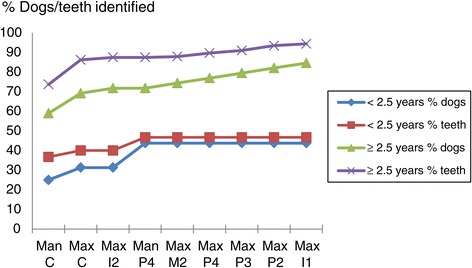
**Identification of presence of clinical attachment loss (CAL) ≥1 mm using partial subset for PPD ≥4 mm.** The portion of the total number of teeth/dogs with CAL ≥1 mm that are identified when successively examining the teeth indicated on the x-axis. Given according to age.

The teeth most likely to present with PPD ≥4 mm were the mandibular canines, and screening of these two teeth would lead to the identification of 57.1% of all dogs and 81.6% of all teeth exhibiting PPD ≥4 mm. Adding the maxillary canines to the partial subset of teeth examined would increase these percentages to 82.1, respectively 94.2%. All dogs with PPD ≥4 mm, and consequently also all teeth so affected could be identified by screening nine out of 21 possible tooth pairs (Table 5). Screening only animals aged over 2.5 years for PPD ≥4 mm had a higher sensitivity than screening younger dogs (Figure 4), but limited sensitivity gains were observed by including other teeth than the canines in the subset of teeth examined. Table 6 shows that examination of the canines would result in the identification of 58.2% of the dogs, and 80.1% of the teeth positive for CAL ≥1 mm. Screening only animals over 2.5 years of age for CAL ≥1 mm had a higher sensitivity than the screening of younger dogs (Figure 5).

**Table 5:** **Hierarchical identification of dogs with recordings of pocket probing depth (PPD) ≥4 mm**

**Tooth- pair examined**	**All dogs (N = 98)**		
	**% PPD positive dogs found**	**% PPD positive teeth found**	NPV* dogs
**Man C**	57.1	81.6	0.64
**+Max C**	82.1	94.2	0.81
**+Max I2**	85.7	95.5	0.84
**+Man P4**	89.3	96.4	0.88
**+Max M2**	92.9	97.3	0.91
**+Max P4**	95.5	97.8	0.93
**+Max P3**	96.4	98.7	0.95
**+Max P2**	98.2	99.6	0.98
**+Max I1**	100	100	1.00

The proportion of the total number of PPD positive dogs, respectively, teeth that are identified by examination of the indicated tooth pairs (sensitivity). Also given for each combination of tooth-pairs is the proportion of test-negative dogs that are truly negative for the presence of PPD ≥4 mm.

*Negative predictive value of screen test at dog level.

**Table 6:** **Presence of clinical attachment loss (CAL) ≥1 mm using the screening subset for pocket probing depth (PPD) ≥4 mm**

**All dogs (N = 98)**		
	**% CAL positive dogs found**	**% CAL positive teeth found**
**Man C**	49.1	69.5
**+Max C**	58.2	80.1
**+Max I2**	60.0	82.1
**+Man P4**	63.4	82.8
**+Max M2**	65.5	83.2
**+Max P4**	67.3	84.7
**+Max P3**	69.1	85.9
**+Max P2**	70.9	88.2
**+Max I1**	72.7	88.9

The proportion of the total number of CAL positive dogs, respectively, teeth that are identified by examination of the tooth pairs indicated for identifying PPD ≥4 mm (sensitivity).

## Discussion

A screening for PD should optimally by a quick, simple and accurate examination of a few teeth, be able to identify diseased dogs. Screened dogs identified as diseased should undergo a full mouth examination for complete diagnosis. Screened dogs identified as non-diseased should safely be regarded as non-diseased, thus eliminating the need for further examination.

This study indicates that examination of the five maxillary tooth pairs P4, P3, P2, I1 and C would identify 85.5% of all dogs and 96% of all teeth with CAL ≥1mm (Table 3), and 58.9% of all dogs and 82.1% of all teeth with PPD ≥4 mm (Table 4). Examination of the five tooth pairs, maxillary I2, M2 and C and mandibular C and P4 would identify 92.9% of all dogs and 97.3% of all teeth with PPD ≥4 mm (Table 5), and 65.5% of all dogs and 83.2% of all teeth with CAL ≥1mm (Tables 6). These results indicate that a substantial part of teeth with CAL ≥1mm and PPD ≥4 mm among Beagle dogs may be found by means of a relatively simple screening including the maxillary P4, P3, P2, I1 and C if CAL ≥1 mm is the clinical parameter of interest or all C’s, maxillary I2, M2 and mandibular P4 if PPD ≥4 mm is the clinical parameter of interest. Before screening for either CAL or PPD a decision has to be made whether the goal is to identify periodontally diseased animals or teeth or to estimate treatment needs. If treatment planning is the goal then the subset of teeth screening for PPD ≥4 mm should be used, if identification of animals or teeth with PD is the goal then the subset of teeth screening for CAL ≥1mm should be used. However, it should be borne in mind that the results presented here clearly only pertain to the present study population. In previous studies, certain tooth types have been found to be more prone to CAL or PPD. In poodle dogs the teeth most often found to be “periodontally involved” were the canines and maxillary P4 [17]. In a study of different breeds the canines were observed as the teeth most often exhibiting loss of attachment while teeth exhibiting severe CAL more frequent were the maxillary P4 and molars [16]. Deep periodontal pockets were most often found in the canines, incisors and maxillary P4 in small breeds [18], but in another study of Beagles it was the mandibular P4 [24]. Finally, a more recent study in a population of pet dogs; found that maxillary teeth were more frequently affected by PD compared to mandibular teeth [25]. The screening protocol suggested here thus, should be tested in other dog populations before the partial recording system can be considered a valid tool for screening for PD across different breeds.

The more teeth that are included in a subset of teeth to be examined, the more information will be obtained, and the closer the results will come to the true situation as seen in a full-mouth examination. The main cost of this information is anaesthesia time. However, when using a partial examination some loss of information has to be accepted. The question is how comprehensive the loss may be, compared to the time spent on the screening. If a diseased dog is tested negative by the screening and therefore does not undergo a full mouth, site specific examination, some affected teeth or sites, different from the ones of the screening, may not be diagnosed. Our determination of the “best” subset of teeth to be examined is necessarily based on some degree of subjective assessment.

In conducting this study we have made two assumptions, the reasonability of which may be discussed. We have considered all measurable CAL (i.e., ≥1 mm), where it is commonplace in the human periodontal literature to consider attachment loss as a sign of periodontitis only when it exceeds 3 mm (i.e., ≥3 mm). However, the latter decision is entirely arbitrary and not evidence based [26,27], and will lead to underestimation of PD. It is our view that this is gradually being recognized. Concerning PPD there is no natural 0-point, and we therefore have arbitrarily chosen a cut-off-point of 4 mm as the threshold for a positive diagnosis. This threshold corroborates the threshold commonly used in the human clinic, but could certainly be discussed in the context of a dog population, particularly as the human threshold was chosen as the depth at which tooth brush bristles can no longer reach sufficiently into the pocket to clean the teeth. Further; natural cleaning mechanisms are believed to be impaired in pockets with PPD ≥4 mm leaving these animals with a need for detailed periodontal treatment planning. However, the relatively few teeth that appeared with pockets ≥4 mm among the younger dogs support our contention that we have not applied an unduly liberal definition of what constitutes a “pathological” pocket depth among dogs.

## Conclusions

This screening protocol is suitable for examination of larger groups of laboratory Beagle dogs and our findings indicate that diseased dogs are identified with a high degree of sensitivity. Tooth pairs screening for CAL ≥1 mm included the maxillary P4, P3, P2, I1, C and tooth pairs screening for PPD ≥4 mm included the maxillary I2, M2, the mandibular P4 and both maxillary and maxillary C. This procedure will typically take a few minutes. The sensitivity of the screening is higher when only dogs older than 2.5 years are screened. Possible breed differences in predispositions to periodontal disease may exist. Before this screening can be used in clinical practice, it has to be validated in canine breeds other than Beagle dogs and in populations with larger age variation.

## Abbreviations

C: Canine tooth
CAL: Clinical attachment loss
I1,2,3: Incisor tooth1,2 or 3
M1,2,3: Molar tooth 1,2 or 3
Man: Mandibular
Max: Maxillary
NPV: Negative predictive value
P2,3,4: Premolar tooth 2,3 or 4
PD: Periodontal disease
PPD: Pocket depth
SD: Standard deviation
